# Modulation of vulvovaginal atrophy (VVA) by Gelam honey in bilateral oophorectomized rats

**DOI:** 10.3389/fendo.2023.1031066

**Published:** 2023-02-27

**Authors:** Nur Hilwani Ismail, Siti Fatimah Ibrahim, Mohd Helmy Mokhtar, Azyani Yahaya, Aini Farzana Zulkefli, Sheril June Ankasha, Khairul Osman

**Affiliations:** ^1^ Faculty of Applied Sciences, School of Biological Sciences, Universiti Teknologi MARA, Shah Alam, Malaysia; ^2^ Department of Physiology, Faculty of Medicine, Universiti Kebangsaan Malaysia, Kuala Lumpur, Malaysia; ^3^ Department of Pathology, Faculty of Medicine, Universiti Kebangsaan Malaysia, Kuala Lumpur, Malaysia; ^4^ Centre of Diagnostic, Therapeutic & Investigative Studies, Faculty of Health Sciences, Universiti Kebangsaan Malaysia, Bangi, Malaysia

**Keywords:** vulvovaginal atrophy, genitourinary syndrome, menopause, Gelam honey, reproductive health, nutraceuticals, supplement, bilateral oophorectomy

## Abstract

**Introduction:**

Vulvovaginal atrophy (VVA) is a common condition in post-menopausal women. Symptoms of VVA include dyspareunia, vaginal dryness, vaginal and/or vulvar itching, burning and soreness, dysuria and vaginal bleeding accompanying sexual activity. These symptoms are physiological responses to hypoestrogenicity, inducing atrophy of the vagina epithelia and sudden reduction in mucous production. Prevailing therapy for VVA is hormone replacement therapy (HRT), notably estrogen, progesterone or a combination of the two. However, using HRT is associated with an increased incidence of breast and endometrial cancer, venous thromboembolism in the lungs and legs, stroke and cardiovascular complications.

**Methods:**

This study evaluated Malaysian Gelam honey as a nutraceutical alternative to estrogen HRT (ERT) in alleviating VVA. A total of 24 female 8-weekold Sprague Dawley rats underwent bilateral oophorectomy. A minimum of 14 days elapsed from the time of surgery and administration of the first dose of Gelam honey to allow the female hormones to subside to a stable baseline and complete recovery from surgery. Vaginal tissues were harvested following a 2-week administration of Gelam honey, the harvested vagina tissue underwent immunohistochemistry (IHC) analysis for protein localization and qPCR for mRNA expression analysis.

**Results:**

Results indicated that Gelam honey administration had increased the localization of Aqp1, Aqp5, CFTR and Muc1 proteins in vaginal tissue compared to the menopause group. The effect of Gelam honey on the protein expressions is summarized as Aqp1>CFTR>Aqp5>Muc1.

**Discussion:**

Gene expression analysis reveals Gelam honey had no effect on Aqp1 and CFTR genes. Gelam honey had up-regulated Aqp5 gene expression. However, its expression was lower than in the ERT+Ovx group. Additionally, Gelam honey up-regulated Muc1 in the vagina, with an expression level higher than those observed either in the ERT+Ovx or SC groups. Gelam honey exhibits a weak estrogenic effect on the genes and proteins responsible for regulating water in the vaginal tissue (Aqp1, Aqp5 and CFTR). In contrast, Gelam honey exhibits a strong estrogenic ability in influencing gene and protein expression for the sialic acid Muc1. Muc1 is associated with mucous production at the vaginal epithelial layer. In conclusion, the protein and gene expression changes in the vagina by Gelam honey had reduced the occurrence of vaginal atrophy in surgically-induced menopause models.

## 1 Introduction

Vulvovaginal atrophy (VVA) is associated with the changes in anatomical and functionality of vulvo-vaginal tissues occurring with menopause and aging with a plethora of conditions related to the genitals, sexual intimacy and urinary symptoms (1). It is a chronic and progressive condition resulting from hypoestrogenicity, a hallmark of menopause due to the cessation of ovarian estradiol secretion leading to reduced blood flow to the vagina and clitoris and ultimately induces atrophy of vagina epithelia (2). The pathogenesis of VVA is due to epithelial thinning, changes in smooth-muscle cell function, reduction in the content of collagen elastin and hyaluronic acid, and an increase in the density of connective tissues as well as avascularity. These characteristics would lead to atrophy of the vagina epithelia and, consequently a significant reduction in mucous production. These physiological changes are detrimental to sexual function and the quality of life of menopausal women (3). Sexually active menopausal women commonly complain of genital symptoms (dryness, itching, burning, irritation, bleeding and discharge of vagina), urinary symptoms (dysuria, urgency, frequency, nocturia, urinary incontinence and recurrent urinary tract infections), sexual complication symptoms (dyspareunia, light bleeding and decreased vaginal lubrication during coitus) together with vaginal dryness (1, 4). Among all menopausal complaints, vaginal dryness is the most common symptom reported by post-menopausal women in surveys (5, 6) and in clinical studies (7, 8). The occurrence of vaginal dryness in women is prevalent across all three stages of menopause: pre-, peri- and postmenopausal women at a prevalence rate of between 20% to 40% (9). The female hormones estrogen and progesterone’s secretion influence the cellular architecture of the female reproductive tract by tissue thickening and mucus production in both the uterus and vagina (10, 11). The presence of estrogen typically produces mucus with a low viscosity and a higher pH value such as in the case during ovulation. On the other hand, progesterone secretion produces mucus which is highly viscous and has a lower pH value, predominantly observed in the luteal phase of the menstrual cycle (12). In the vagina, the non-keratinized epithelium of stratified squamous cells is protected by the cervico-vaginal mucosal layer comprised of secreted mucus (13). Mucus is the gel secretion from the epithelial layer for example of the female reproductive tract and is predominantly composed of water, ions and solid matter. Mucin glycoproteins, proteoglycans, lipids, defense proteins and lysozyme forms the solid matter components (14). Furthermore, mucin glycoproteins, which constitutes of proteins, sugar and sialic acid, is the component that exhibits the viscoelastic properties of the mucus (15). Mucins are primarily composed of long and heavily glycosylated macromolecules with proline, threonine and/or serine residues. Mucins are usually 10-40 MDa in size (15) and functions in modulating and maintaining the structural and functional integrity of the vagina (16, 17). It forms a protective gel in the female reproductive tract and is part of the mucousal layer that protects the female reproductive tract (18). In normal healthy women, the mucus protects the female reproductive tract by lubrication and hydration of the epithelial surfaces and prevents trauma from large shear forces during copulation. The mucosal defense to the epithelial surfaces hinders microbial adherence and epithelial invasion as well as mediation of bacterial eradication due to its dense microstructure (10, 14, 19, 20). Steroid hormones regulate the biochemical and biophysical properties of mucus. Estrogen principally determines the volume and quality of mucus depending on the phase of the menstrual cycle and immediate health condition of the individual (21, 22).

The regulation of the fluid environment in the female reproductive tract is of physiological importance for multiple events relating to reproduction. Variations of the normal volume or compositions of the reproductive tract fluid are governed mainly by fluid movements across the epithelia (23). The fluid movements across the epithelia are consequential to solute movement, particularly Na+ and Cl- ions. Aquaporins (Aqp) and Cystic Fibrosis Transmembrane Conductance regulator (CFTR) play important roles in fluid environment homeostasis and mucus viscosity (24, 25). Electrolyte homeostatis and fluid absorption and secretion are dependent on the transport of Na+ and Cl- ions. The movements of these ions affect water transport from the lumen into the blood or from the plasma into the lumen (26, 27). Estrogen exhibits a vasodilating effect on the genitals and may induce increased blood flow and facilitate transport of vaginal fluid through the epithelium by modulation of neurotransmitters and aquaporins (Aqps) (28). Hence, it is highly suggested that Aquaporins are responsible for the water movement at the epithelial layer and contribute to the water component of the vaginal mucus. Aqps are a family of transmembrane channel proteins that expedites transport of solutes and water across the membrane. There are 13 subtypes of identified Aqps that are categorized based on their physiological functions. The three categories are: (i) Aqps for selective permeation of water (ii) aquaglyceroporins which are Aqps that allow permeation of water plus glycerol and (iii) other Aqps that have functions other than the two previously described. Aqp1 and Aqp5 are categorized as Aqps that are selectively permeable to water. Aqp1 have been reported to be downregulated in instances of oxidative stress such as in the case of diabetes mellitus (9). The passive transport of water *via* Aqp following the direction of osmotic pressure which is dependent on solute concentration, maintains the volume of the fluid environment. The Na+ absorptive and Cl- secretion transporting mechanism in the female reproductive tract is present in both the oviduct and endometrial epithelia of humans and murines. CFTR has been implicated in this transport mechanism and found to affect exocrine glands and secretory responses regulated by neuro-hormonal changes in the female reproductive tract (25, 29).

The treatment for VVA primarily is to alleviate the symptoms *via* the lubrication and functional maintenance of the epithelial lining. Prevailing therapy for VVA can either be non-hormonal therapy or hormone replacement therapy (HRT) such as estrogen, progesterone or a combination of the two. The non-hormonal therapy, the first to be prescribed to patients, consists of vaginal lubricants and moisturizers. It includes using short-acting substances that are sufficient for patients with very mild complaints by improving vaginal dryness-related symptoms (30). Hormone replacement therapy (HRT) is considered as the “gold standard” in menopausal treatment. It is the pharmacologic therapy with local estrogen, progesterone or a combination of the hormones (31, 32). However, long-term use of HRT is associated with the risks of endometrial cancer, invasive breast cancer, thromboembolic events, and abnormal uterine bleeding (33–35). These issues have thus led to a search for a more natural complement or alternative to HRT in preventing severe VVA. Natural alternatives refer to non-hormonal methods to relieve menopausal symptoms (35). Nutraceutical alternatives such as honey, that are used in Apitherapy, could hold the potential as an alternative to estrogen HRT (ERT) in alleviating VVA. Apitherapy is a branch of complementary therapy that uses natural honeybee products such as honey as therapeutic agents (36–39). Honey is a complex biological material composed of sugar compounds, enzymes, minerals and antioxidants. These compounds collectively contribute to their anti-inflammatory, broad-spectrum antimicrobial, anti-cancer and immunomodulatory properties (36, 40–42). Gelam honey is multifloral honey produced by *Apis dorsata* bees in beehives formed in Gelam trees (Malaleuca sp.) deep in Malaysia’s rainforest (43, 44). The therapeutic attributes of honey are as old as human civilization and predate ancient Greece, Rome and Egypt. Honey is frequently referenced in sacred and religious texts as well as in the traditional medicine contexts of Chinese, Ayurveda together with Arabic, Galenic, Yunani and Malay traditional medicine (45, 46). It is essential to note that nutraceuticals are vulnerable to adulteration and prove a challenge to standardize (47, 48). To circumvent this problem, Gelam honey used in this research was standardized based on a Gelam honey physicochemical characteristics reference value we have previously proposed for Malaysian Gelam honey (38).

A preliminary study on the dose-response effects of Gelam honey administration (0.2-8g/kg bw/day) on normal sexually matured female SD rats showed potential for attenuation of uterine and vaginal atrophies (38). Gelam honey favored the thickening of the endometrial stroma and endometrial surface endothelial layer. However, the effects observed both in the vaginal epithelia and vaginal squamous epithelia were not as promising as the observation in the uterine tissues. The vaginal epithelium became thinner and the squamous epithelium, which makes up the mucosal layer, became less pronounced with increasing dosage of Gelam honey. We concluded that the absolute effect of Gelam honey on both the vaginal and uterine tissue could be observed in the menopause model. Here, we conducted a controlled experiment with bilateral oophorectomized rats as a menopause model to evaluate the mechanism of Gelam honey in VVA modulation. The study was designed to identify the potential of Gelam honey on attenuating the effects of menopause on serum estrogen, progesterone and testosterone levels and also the effects of Gelam honey on the expression of genes and proteins responsible for cervico-vagina mucous production. The serum hormone secretion and expression levels of the four genes and proteins are important indicators for mucous production in the female reproductive tract for assessment of atrophy in the vaginal epithelia.

## 2 Materials and methods

### 2.1 Ethical approval of experimental animals

The experimental animals were obtained from UKM’s institutional Laboratory Animal Research Unit (LARU), Faculty of Medicine, UKM. The ethical review and approval of the study protocol for handling, treatment and euthanisation of experimental animals were awarded by Universiti Kebangsaan Malaysia Animal Ethics Committee (UKMAEC) for animal research (approval no.: FISIO/PP/2019/SITI FATIMAH/30-OCT./1059-OCT.-2019-APR.-2022-NAR-CAT2).

### 2.2 Experimental animals

This study used 24 female 6-week-old Sprague Dawley rats (RRID : MGI:5651135) weighing 180-210 g. All experimental animals were acclimatized for two weeks before commencement of the study to ensure sexual maturity at eight weeks old. The rats were housed in individually ventilated cages (IVC) on Level 8, Faculty of Medicine Pre-Clinical Building, UKMMC. The IVC cages were kept under artificial lighting of 12-h light/12-h dark conditions at the animal unit, in an air-conditioned room at 25 ± 2°C with 50–55% relative humidity. Animals were given standard food pellets and water *ad libitum*.

### 2.3 Experimental schedule and design

**Figure 1:**
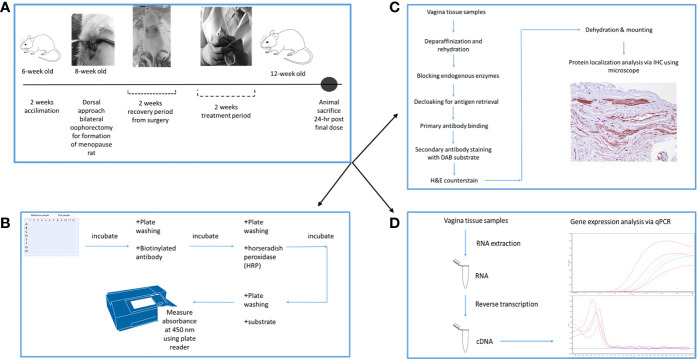
Experimental schedule and design of the experiment. **(A)** is the timeline for the experiment, depicting the period of bilateral oophorectomy surgery and treatment period. **(B)** is the protocol flowchart for ELISA estrogen hormone analysis. **(C)** is the protocol flowchart for protein localization in the vaginal tissues. **(D)** is the protocol flowchart for the mRNA expression analysis in the vaginal tissues using qPCR.

The 24 rats were randomly assigned to 4 groups. Gelam honey (Gelam honey +Ovx), Estrogen replacement therapy group (ERT+Ovx) and menopause control (Ovx) group all underwent bilateral oophorectomy for complete removal of both ovaries. Rats in the Sham control group (SC) underwent similar surgical stress. However, both ovaries remained intact. Upon recovery from surgery, animals were given the respective treatments depending on the study group. Gelam honey group received 0.2 g Gelam honey/kg bw/day. The estrogen replacement therapy group (ERT) received 3µg 17-β estradiol/kg bw/day to mimic estrogen hormone replacement therapy. The dosage is based on the guidelines as outlined in the TG 440 Uterotrophic Bioassay guidance document (49). The Ovx and SC groups received only distilled water. **Figure 1** is the schematic of the experimental schedule and design.

### 2.4 Bilateral oophorectomy surgery (Ovx)

The standard menopause model for the rat was achieved *via* complete removal of both ovaries by means of bilateral oophorectomy surgery. The surgery was conducted on 8-week-old anaesthetized rats given a cocktail of Ketamine/xylazine. This was done through IV injection at a dose of 0.1 mL/100 g bw equivalent to 91 mg/kg Ketamine: 9.1 mg/kg xylazine for 60-80 min anesthesia (50). The surgery was based on a method by Murakami et al. (51), with slight modifications. Rats were placed in a dorsal position, and a 2 cm x 3 cm surgical area was shaved above the lower back. A 1 cm incision is made at a distance of 1 finger-width apart from the left of the spine to enter the abdominal cavity and expose the ovary and oviduct. Ligation of the ovary is achieved 3 mm below the ovaries using absorbable sutures, size 4-0 monofilament Vicryl^®^ (Ethicon Inc., New Jersey, USA) made with an uninterrupted suturing technique. Excisional biopsy is conducted by complete removal of the ovary followed by closure with a non-absorbable nylon suture with a 16 mm 3/8^th^ circle reverse-cutting eye (Unik Surgical Sutures Manufacturing Co., Taiwan) using an interrupted suturing technique. The same procedure is repeated for removal of the right ovary with an initial 1 cm incision made at a distance of 1 finger-width apart from the right of the spine. Bilateral oophorectomy was performed on all rats in Gelam honey +Ovx, ERT+Ovx and SC groups. Rats in the Sham control group underwent similar surgical stress. However, both ovaries remained intact. Following surgery, all animals were given 0.1 mL Kombitrim antibiotic (Kombivet, Netherlands) *via* intramuscular injection. Vagina smear cytology was conducted for two weeks to ensure the surgery was successful and rats did not regress to the estrus phase.

### 2.5 Gelam honey treatment

Rats that underwent bilateral oophorectomy surgery were allowed to recover for two weeks before commencement of Gelam honey treatment. This permitted the hormones to regress to a stable baseline and complete recovery from surgery (OECD TG 440, 2007). Gelam honey at a dose of 0.2 g/kg bw per day was given *via* oral gavage for two–weeks using an 18-G tube.

### 2.6 Serum estrogen hormone profile

The rat blood through retro-orbital bleeding was collected into serum separator tubes (BD Vacutainer SST™, Becton Dickinson, Franklin Lakes, NJ, USA). Blood was allowed to clot at room temperature before centrifugation at 3000 x g for 15 min. The serum estrogen hormone levels were analyzed using a competitive enzyme-linked immunosorbent assay (ELISA) according to the manufacturer’s guidelines (Elabscience, Wuhan, China). In brief, the biotinylated antibody solution and reference standards were added into the reference wells of the ELISA plate. On the other hand, biotinylated antibody solution and serum samples were added to the test wells of the ELISA plates. Analysis of reference samples and test samples were conducted in duplicates. The filled ELISA plates were sealed and incubated for 45 min at a temperature of 37°C. At the end of incubation, plate washing was conducted by aspirating the solution from each well and soaking it with the wash buffer for 1-2 min before discarding the wash buffer and pat-drying the plate onto a paper towel. Plate washing step was repeated three times. Next, the wells were incubated with horseradish peroxidase (HRP) conjugate for 30 min at 37°C. This step was followed by a plate washing process repeated five times. The following stage is the substrate-conjugating stage. During this stage, components for the analysis is light-sensitive. Therefore, the ELISA plate was protected from over-exposure to light. In this satge, the substrate reagent was added to each well and the plate was incubated for 15 min at 37°C. At the end of the incubation period, stop solution was added to each well to stop the reaction and the absorbance of the solution in the wells was immediately measured at 450 nm using a microplate reader (SpectraMax Plus 384 Microplate Reader, Molecular Devices, California, USA). Quality control was achieved by ensuring that the stop solution was added in the same order in which the substrate solution was added. A standard curve was generated using the absorbance value of reference sample which are the standard dilutions of the hormone. The absorbance value recorded from the reading of test sample was used to calculate the exact amount of estrogen hormone present in each sample by comparing it to the standard curve.

### 2.7 RNA extraction & reverse transcription

Vaginal tissue samples were stored at −80°C until further use in the RNA extraction stage. Maximum weight of 30 mg of vagina tissue was used as starting material for every RNA extraction procedure. Mechanical lysis of frozen vagina tissue samples was achieved by pouring liquid nitrogen over the tissue and crushing the tissue until powder using a pestle and mortar. The powdered vagina tissue was then used for the homogenization stage of RNA extraction using GF-1 Total RNA Extraction Kit (Vivantis, Malaysia) according to the manufacturer’s protocol. RNA samples were treated with DNAse I to remove traces of genomic DNA contamination. Total RNA was then transcribed to cDNA (10 μg of RNA in 20 μL reaction) using Viva cDNA Synthesis Kit (Vivantis, Malaysia) with a final cDNA concentration of 50 ng/μL.

### 2.8 Quantitative real-time PCR (RT-qPCR)

Vagina cDNA was mixed with ViPrimePLUS Taq qPCR Green MasterMix I SYBR^®^ Green Dye (Vivantis, Malaysia) and specific primer pairs as listed in **Supplementary Table S1**. Reverse transcription was performed in a 10 µl reaction volume on the CFX96™ Real-Time System (Bio-Rad) with a CFX Manager software (Bio-Rad) to record the cycle threshold (Ct) values. A no-template control (NTC) and a no-reverse transcriptase (-RT) control in the absence of reverse transcriptase were used as controls for the experiment. PCR reactions were performed in triplicates. The following qPCR cycling protocol was used: 95°C for 2 min, 95°C 15 s, 60°C 60 s, 40 cycles in total and a melt curve at 65-95°C at 0.5°C increment per step at 10 s per step. The expression levels of target genes (*Aqp1, Aqp5, CFTR *and *Muc1*) were normalised against the geometric means of two housekeeping genes: *β-actin* and *Gapdh*. A comparative Ct method (2^-ΔΔCt^) was used to calculate the relative gene expression values. All vagina tissue mRNA levels of Gelam honey-treated post-menopausal rats were expressed as fold changes relative to mRNA levels of the menopausal negative control rats and ERT-receiving positive control rats.

### 2.9 Tissue processing and embedding

Tissue samples were preserved in 10% neutral buffered formalin prior to tissue processing, tissue embedding and microtomy before immunohistochemistry staining for protein localization.

#### 2.9.1 Tissue processing

The harvested tissues were processed with the following reagents and respective soaking duration. The stages are formalin, 3.5 hrs; formalin, 0.5 hr; six ascending gradient of alcohols (75%, 90%, 95%, 100%, 100%, 100%), each step for 1 hr; three changes of xylene at 1 hr per step and lastly wax infiltration in three changes of wax at 1.5 hrs per step. This stage was performed using Excelsior™ AS tissue processor (Thermo Scientific™, USA).

#### 2.9.2 Tissue embedding

The processed organs are then embedded in paraffin wax to make blocks of tissue samples on HistoStar™ embedding workstation (Thermo Scientific™, USA) and cooled on a Leica EG1150 C cooling plate (Leica Biosystems, Germany).

#### 2.9.3 Microtomy

The paraffin blocks of tissue samples were sectioned into 5 µm sections for use in immunohistochemistry staining on Leica RM2245 microtome (Leica Biosystems, Germany). The ribbon of the sections were floated in Leica HI 1210 floating bath (Leica Biosystems, Germany) in 40°C water to remove wrinkles before being transferred to Polysine^®^ slides (Thermo Scientific™, USA).

### 2.10 Immunohistochemistry (IHC)

#### 2.10.1 Immunohistochemistry staining

The sections of 5 µm vaginal tissues were rehydrated using two changes of xylene followed by three changes of descending grades of alcohol (absolute ethanol, 80% ethanol and 70% ethanol) before immunohistochemistry (IHC) staining. IHC staining was achieved using mouse and rabbit-specific HRP/DAB IHC Detection Kit-Micro-polymer (Abcam, USA). Following rehydration and quenching of endogenous enzymes with 3% H_2_O_2_, heat-induced epitope retrieval (HIER) was performed using Decloaking Chamber™ NxGen (Biocare, USA) at 110°C for 30 min in pH 6 target retrieval solution (Dako, USA). Upon completion of HIER, slides were blocked for nonspecific background staining using protein block followed by a three times washing using TBS wash buffer (Dako, USA). Then, the slides were fixed with the goat anti-rabbit primary antibody for Aqp1 (Abcam Cat# ab168387, RRID : AB_2810992, Rabbit monoclonal to Aquaporin 1, Abcam, USA), Aqp5 (Abcam Cat# ab78486, RRID : AB_1603410, Rabbit polyclonal to Aquaporin 5, Abcam, USA), mouse monoclonal antibody CFTR (Santa Cruz Biotechnology Cat# sc-376683, RRID : AB_11151574, Santa Cruz Biotechnology, Inc., USA) and mouse-monoclonal antibody Muc1 (Santa Cruz Biotechnology Cat# sc-53381, RRID : AB_628990, Santa Cruz Biotechnology, Inc., USA) proteins. The Aqp1 and Aqp5 antibodies required an additional complementary step of mouse specifying reagent before proceeding with HRP-conjugate incubation for secondary antibody tagging. This is then followed by staining with DAB chromogen and counterstaining with H&E stain (Thermo Scientific, USA) before dehydration and mounting with CoverSeal™-X Mounting Medium (Cancer Diagnostics Inc., USA). To demonstrate the specificity of immunoreaction, negative and positive controls were performed for all immunoreactions. For immuno-negative controls the primary antibody at similar dilutions was replaced with normal rat serum. For immuno-positive controls, the following tissues were tested: rat kidney was used for Aqp1, Aqp5 and CFTR while rat lungs were used for Muc1.

#### 2.10.2 Scoring of slides

Stained slides were evaluated by a trainee (N.H.I.) and a pathologist (A.Y.) on an Olympus BX40 Clinical Microscope and imaging software MicroSuite™ system (Olympus America Inc.). Both assessors were blinded to the treatment groups to minimize operator biasness. A minimum of ten fields were examined and photographed for each tissue section using Olympus DP72 Microscope Digital Camera (Olympus America Inc.). The localisation of the four proteins (Aqp 1, Aqp 5, CFTR and Muc 1) was based on a combinative semi-quantitative H-scoring method (52). The percentage area of positively labelled cells (value A) and intensity of IHC reaction (value B) were quantified by the operators, and the product of the two values will give a final score (AxB). In the event of discrepancy, slides were re-evaluated to achieve consensus. The scoring for value A is based on percentage area of positively labelled cells (0 = 0%; 1 = below 30%; 2 = 30-60%; 4 = more than 60%). The scoring for value B is based on intensity of IHC reaction (0 = no reaction; 1 = weak reaction; 2 = mild reaction; 3 = strong reaction).

### 2.11 Measurement of vagina epithelia thickness

Tissue samples underwent tissue processing, tissue embedding and microtomy using similar methods as described in section 2.9. Sections of 5 µm vaginal tissues were deparaffinized using xylene,stained with H&E stain (Thermo Fisher Scientific, Waltham, MA, USA) and dehydration.

#### 2.11.1 Deparaffinization and rehydration

Slides of vagina tissue sections were rehydrated using two changes of xylene followed by three changes of descending grades of alcohol (absolute ethanol, 80% ethanol and 70% ethanol) and water. The slides were incubated for 3 min at each respective stage. Tissue sectiones that were processed were then used for H&E staining.

#### 2.11.2 H&E staining

Deparaffinised and rehydrated slides were stained in hematoxylin for 3 min. This was followed by rinsing in tap water, incubating in clearing solution, second rinse in tap water, incubation in Blueing solution followed by third rinse in tap water. The tissue sections were processed at each stage for 3 min respectively. Finally, tissue sections were counterstained with eosin for 30 sec followed by a final rinse in tap water for 30 sec. H&E stain used was from Thermo Scientific, USA.

#### 2.11.3 Dehydration

The vaginal tissue sections were dehydrated using four changes of ascending grades of alcohol (80%, 90%, 100% and 100%). This was followed by two changes of xylene. Each stage was for 3 min.

Following H&E staining and dehydration, the processed slides were then permanently mounted with Coverseal™-x Mounting Medium (Cancer Diagnostics Inc., Durham, NC, USA), covered with a coverslip. The mean vaginal epithelial thickness was determined from the measurements of ten randomly chosen areas in each section. From each of the ten chosen areas, a total of 20 measurements of vagina epithelial thickness were taken. Slides were observed under a light microscope and micrographs were taken at a 20 × objective using a Olympus BX40 Microscope with a DP27 Olympus camera and a DP-2 SAL processor. Micrographs were further analysed using a computer-aided program (ImagePro Plus v5.0, USA).

### 2.12 Statistical analyses

Results are expressed as mean ± SEM. (standard error of the mean). The statistical analysis was performed with a One-way ANOVA test with a posthoc Tukeys and Dunnette’s test to elucidate differences between the groups (Statistical Package for the Social Sciences version 27.0; SPSS Inc., Chicago, IL). Graphs were presented as mean ± SEM. The level of significance was set at p < 0.05 (*), p < 0.01 (**), and p < 0.001 (***).

## 3 Results

### 3.1 Serum estrogen hormone levels in the vaginal tissue

**Figure 2:**
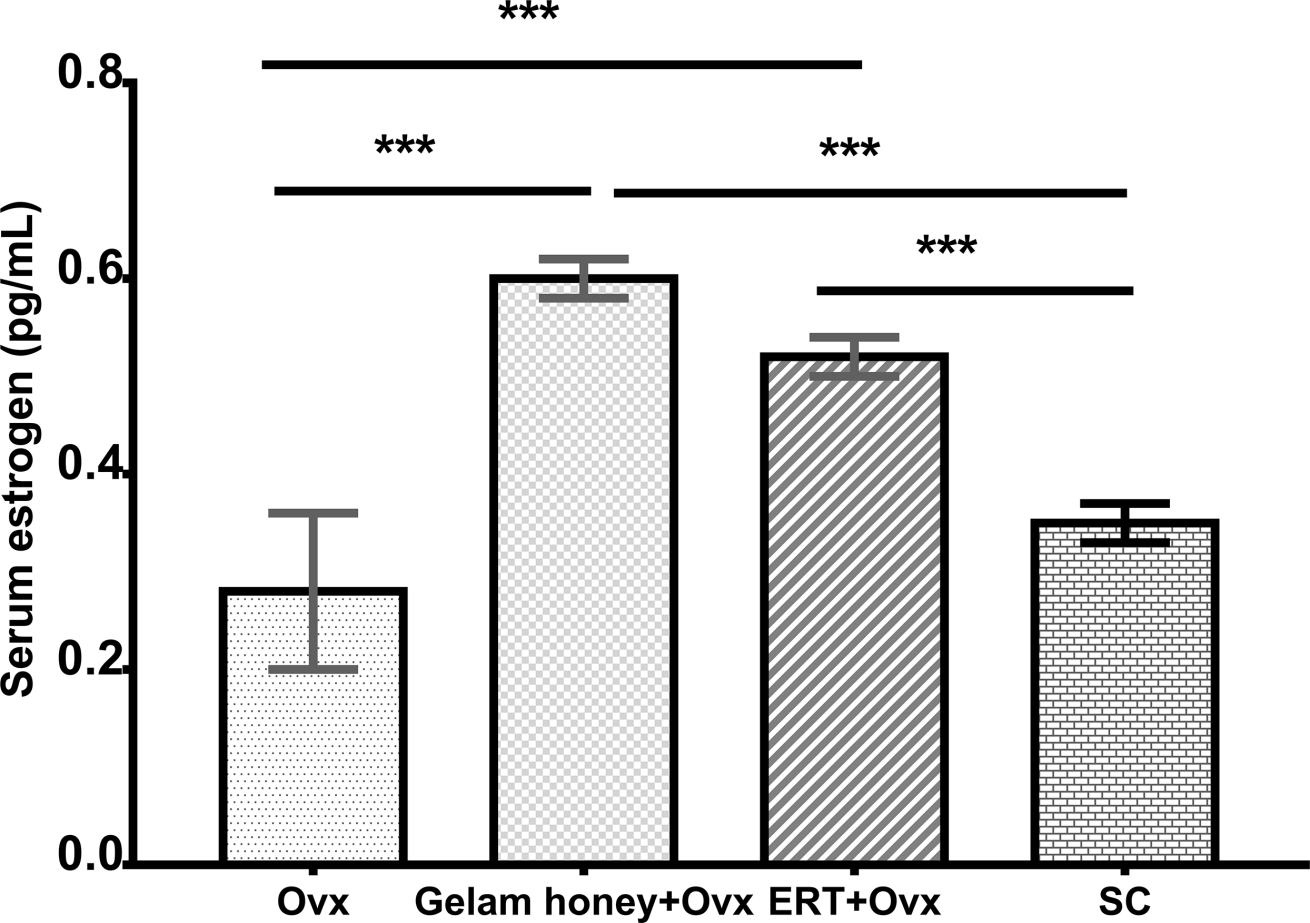
Effect of Gelam honey on estrogen serum levels (pg/mL) in menopause rats. Estrogen serum levels were measured using a competitive enzyme-linked immunosorbent assay (ELISA). All values represent the mean ± SEM, p<0.05. Gelam honey +Ovx: Gelam honey and bilateral oophorectomized group. ERT+Ovx: Estrogen replacement therapy and bilateral oophorectomized group. Ovx: Bilateral oophorectomized group. SC: Sham group.

Gelam honey administration showed a positive effect on the measured serum estrogen levels (**Figure 2**). The menopausal rats benefitted from the estrogenic effect of Gelam honey administration. Gelam honey+Ovx group showed a higher level of serum estrogen than the group receiving estrogen hormone replacement therapy (ERT+Ovx) (p>0.05). The increase in levels of serum estrogen in the menopause model following Gelam honey administration was a significant increase (F (3,17) = 11.940, p < 0.01). Estrogen levels in Gelam honey group (0.59 ± 0.31 pg/mL, p < 0.01) was comparatively higher than Ovx control group (0.28 ± 0.10 pg/mL, p < 0.01) and SC groups (0.35 ± 0.03 pg/mL, p < 0.01). Astoundingly, Gelam honey was as better than ERT in increasing the serum estrogen levels (0.52 ± 0.03, p < 0.667). The observations proves that there is potential in using Gelam honey as an alternative hormone replacement therapy.

### 3.2 *Aqp 1, Aqp5, CFTR* and *Muc 1* mRNA expression in the vaginal tissue

**Figure 3:**
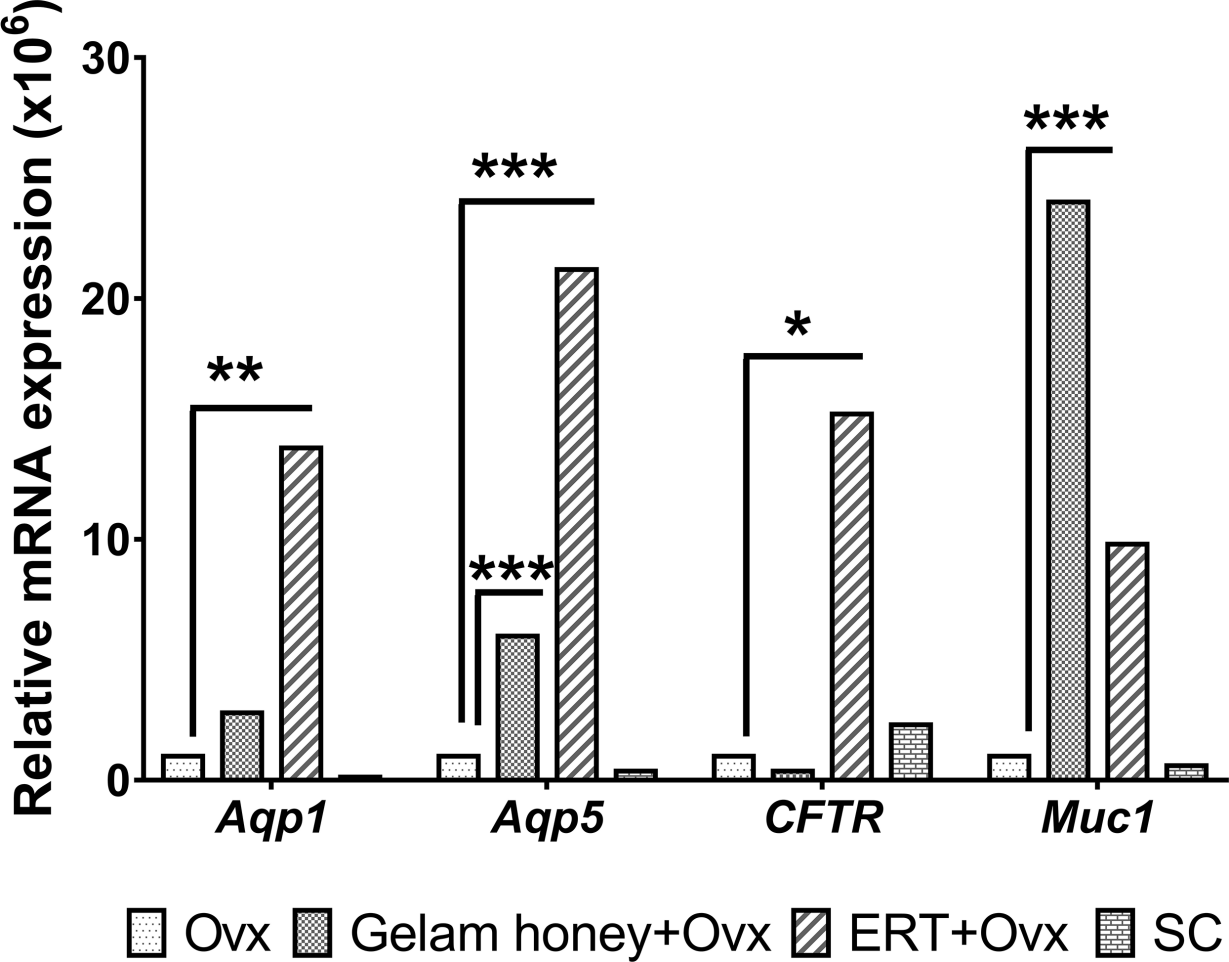
mRNA mean expression for genes *Aqp1, Aqp5, CFTR* and *Muc1* in vagina tissues. The level of significance was set at p < 0.05 (*), p < 0.01 (**), and p < 0.001 (***) Gelam honey+Ovx: Gelam honey and bilateral oophorectomized group. ERT+Ovx: Estrogen replacement therapy and bilateral oophorectomized group. Ovx: Bilateral oophorectomized group. SC: Sham group.

The mRNA expression of genes *Aqp 1, Aqp5, CFTR* and *Muc 1* in vaginal tissues was normalised against Gapdh as the internal control using the ΔΔCt method (53). *Aqp1* expression in the rat vagina tissue showed that rat *Aqp1* expression is estrogen dependent. The *Aqp1* gene was up-regulated in the ERT+Ovx group as compared to the Gelam honey+Ovx group (F (3,15) = 5.186, p < 0.01). Gelam honey administration did not seem to affect *Aqp1* expression in tissues. *Aqp5* expression is also estrogen dependent. Moreover, the expression of *Aqp5* in vagina tissue is up-regulated in the ERT+Ovx group and the Gelam honey+Ovx group F (3,13) = 269.126, p < 0.01. However, ovariectomy *per se* is not detrimental to the expression of *Aqp5*. The *CFTR* gene expression in the vagina tissue was not affected by Gelam honey administration in the Gelam honey+Ovx group. However, *CFTR* gene expression was up-regulated by presence of exogenous estrogen in the ERT+Ovx group F (3,15) = 4.303, p < 0.05 (0.022). Interestingly, the expression of *Muc1* in vagina tissue is independent of estrogen secretion but is positively affected by estrogen presence. Gelam honey administration up-regulated the expression of the *Muc1* gene in vagina tissue compared to other treatments. Furthermore, *Muc1* expression in Gelam honey+Ovx group exceeded the expression levels recorded in the ERT+Ovx group (F (3,14) = 13.970, p < 0.01). **Figure 3** is the overall mRNA expression change of all the respective genes of interest in the vagina tissue.

### 3.3 Aqp 1, Aqp5, CFTR and Muc 1 protein localization in vaginal epithelial tissue

**Figure 4:**
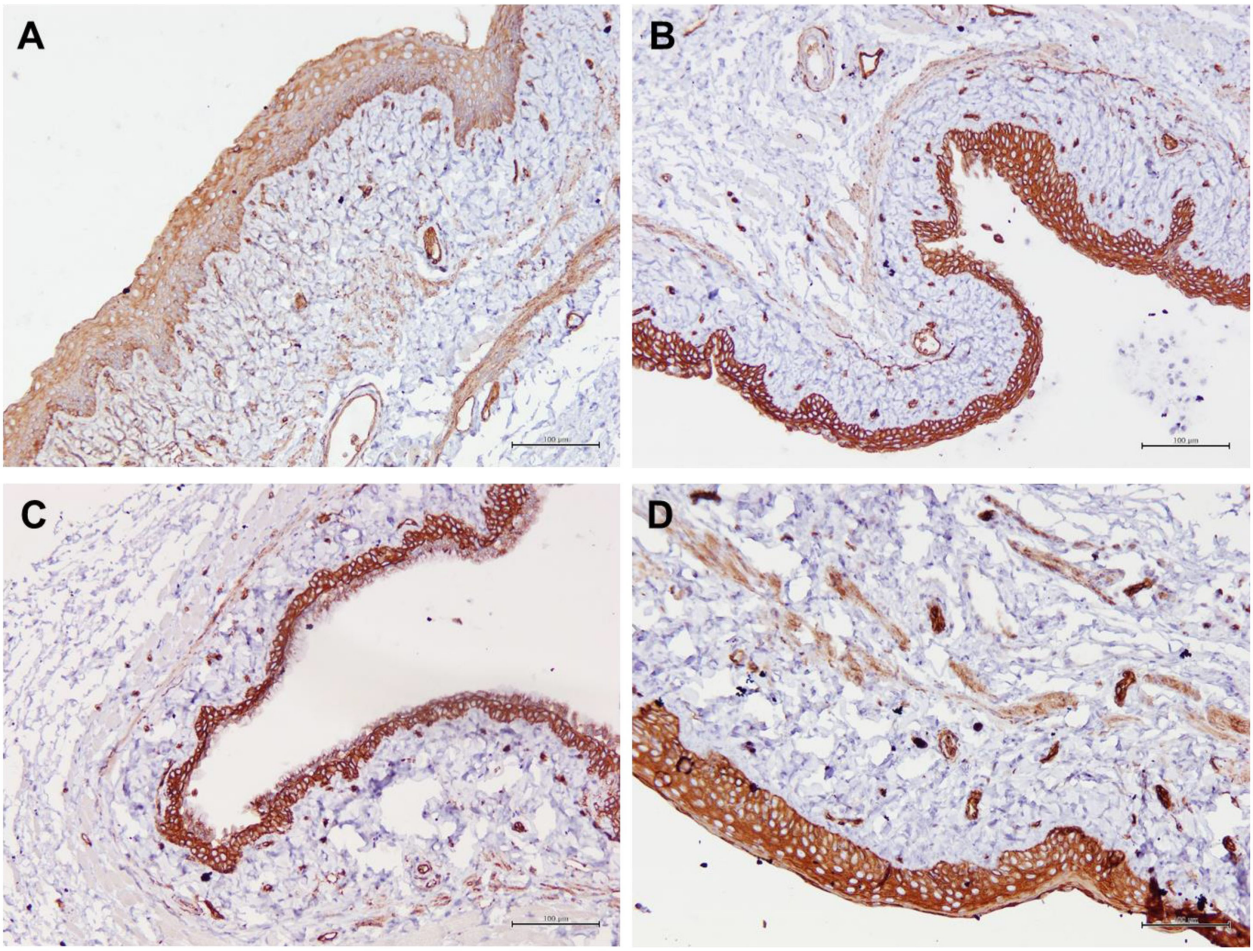
Structure of the rat vaginal epithelium and Aqp1 protein localization in the rat vaginal tissue following a two-week administration of Gelam honey at 0.2 g Gelam honey/kg bw/day doses. **(A)** Gelam honey and bilateral oophorectomized group (Gelam honey+Ovx), **(B)** Estrogen replacement therapy and bilateral oophorectomized group (ERT+Ovx), **(C)** Bilateral oophorectomized group (Ovx) and **(D)** Sham group (SC). The tissues were stained with DAB/HRP stain for protein localization (in brown) and counterstained with H&E for nuclear staining. All measurement bars are equivalent to 100 µm.

**Figure 5:**
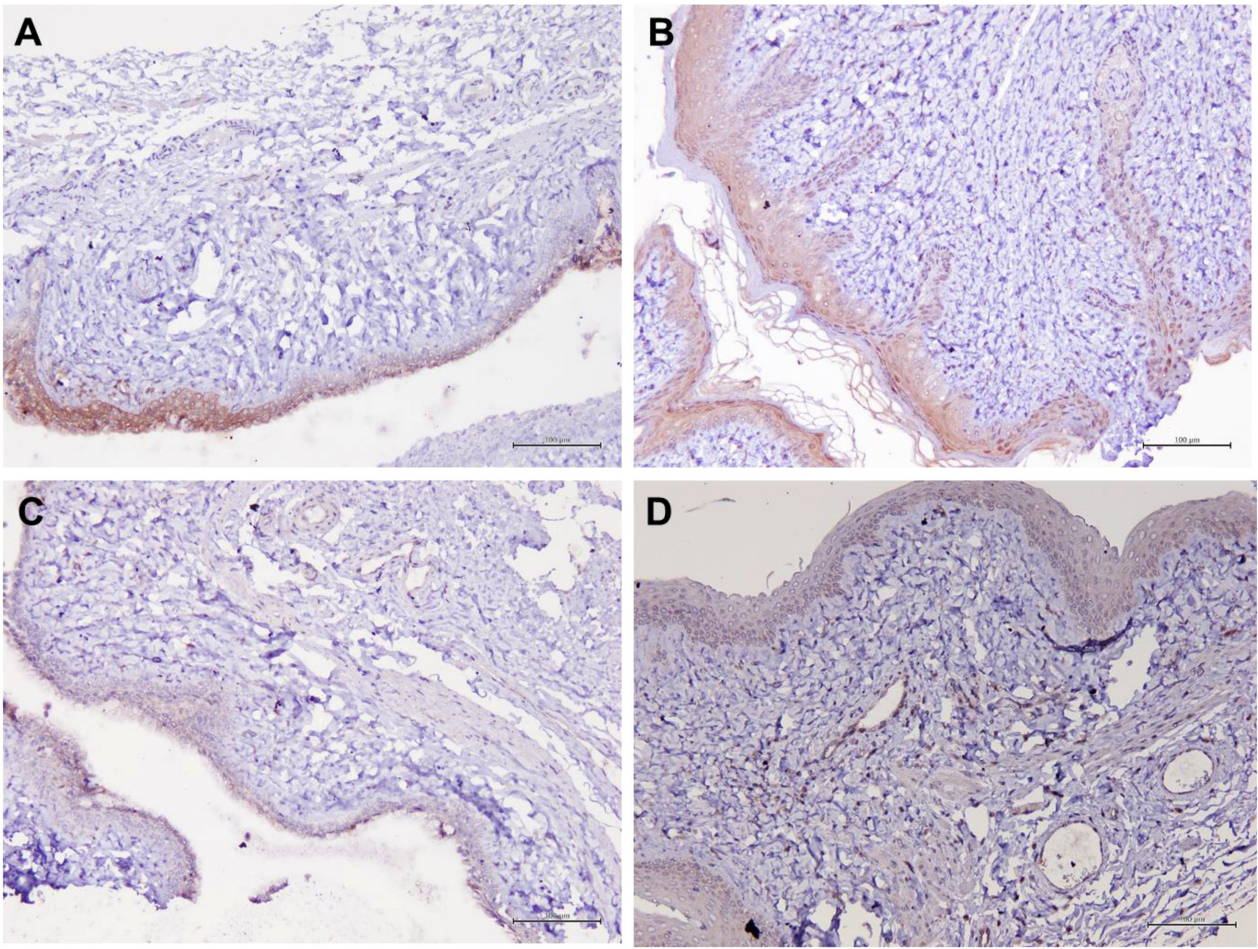
Structure of the rat vaginal epithelium and Aqp5 protein localization in the rat vaginal tissue following a two-week administration of Gelam honey at 0.2 g Gelam honey/kg bw/day doses. **(A)** Gelam honey and bilateral oophorectomized group (Gelam honey+Ovx), **(B)** Estrogen replacement therapy and bilateral oophorectomized group (ERT+Ovx), **(C)** Bilateral oophorectomized group (Ovx) and **(D)** Sham group (SC). The tissues were stained with DAB/HRP stain for protein localization (in brown) and counterstained with H&E for nuclear staining. All measurement bars are equivalent to 100 µm.

**Figure 6:**
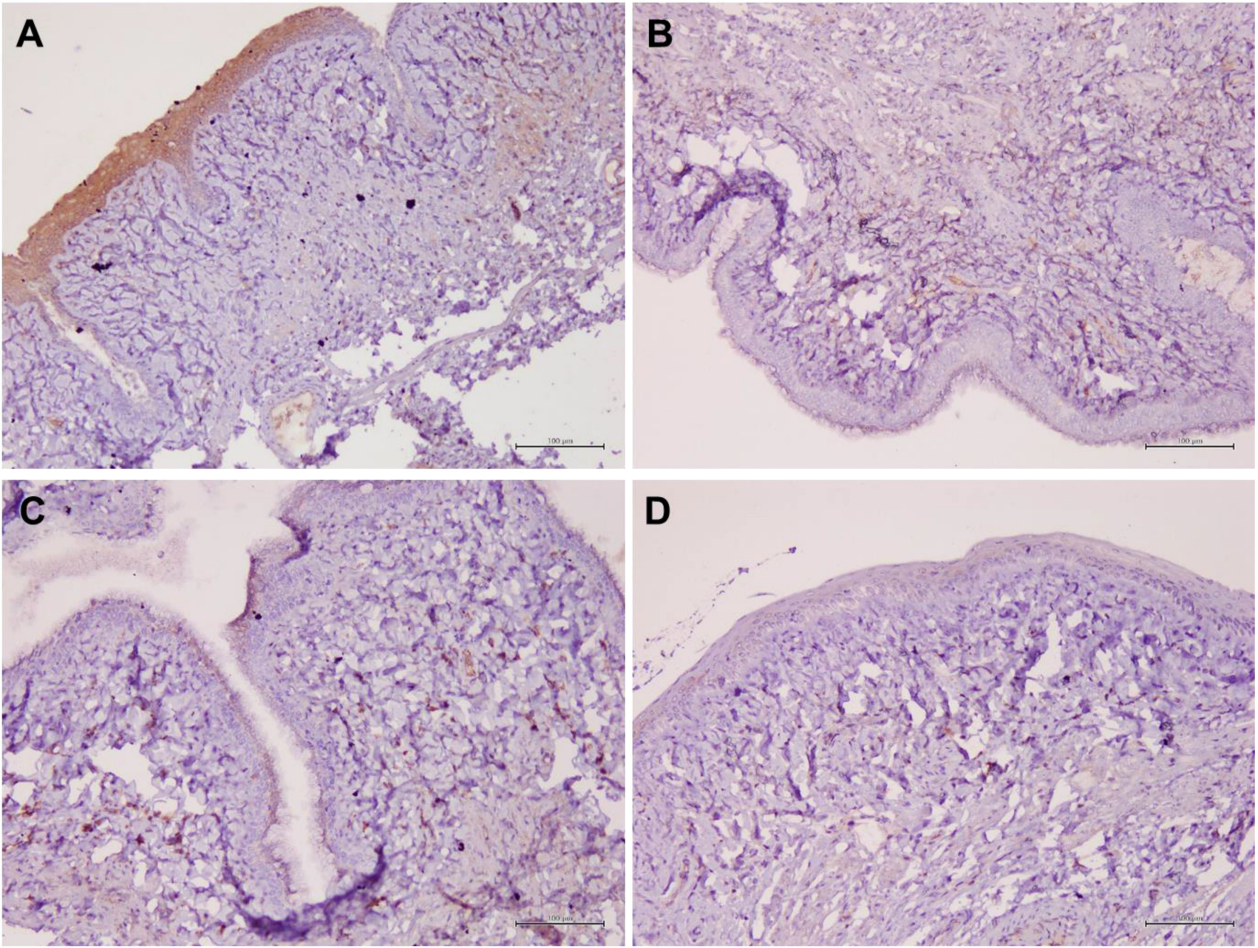
Structure of the rat vaginal epithelium and CFTR protein localization in the rat vaginal tissue following a two-week administration of Gelam honey at 0.2 g Gelam honey/kg bw/day doses. **(A)** Gelam honey and bilateral oophorectomized group (Gelam honey +Ovx), **(B)** Estrogen replacement therapy and bilateral oophorectomized group (ERT+Ovx), **(C)** Bilateral oophorectomized group (Ovx) and **(D)** Sham group (SC). The tissues were stained with DAB/HRP stain for protein localization (in brown) and counterstained with H&E for nuclear staining. All measurement bars are equivalent to 100 µm.

**Figure 7:**
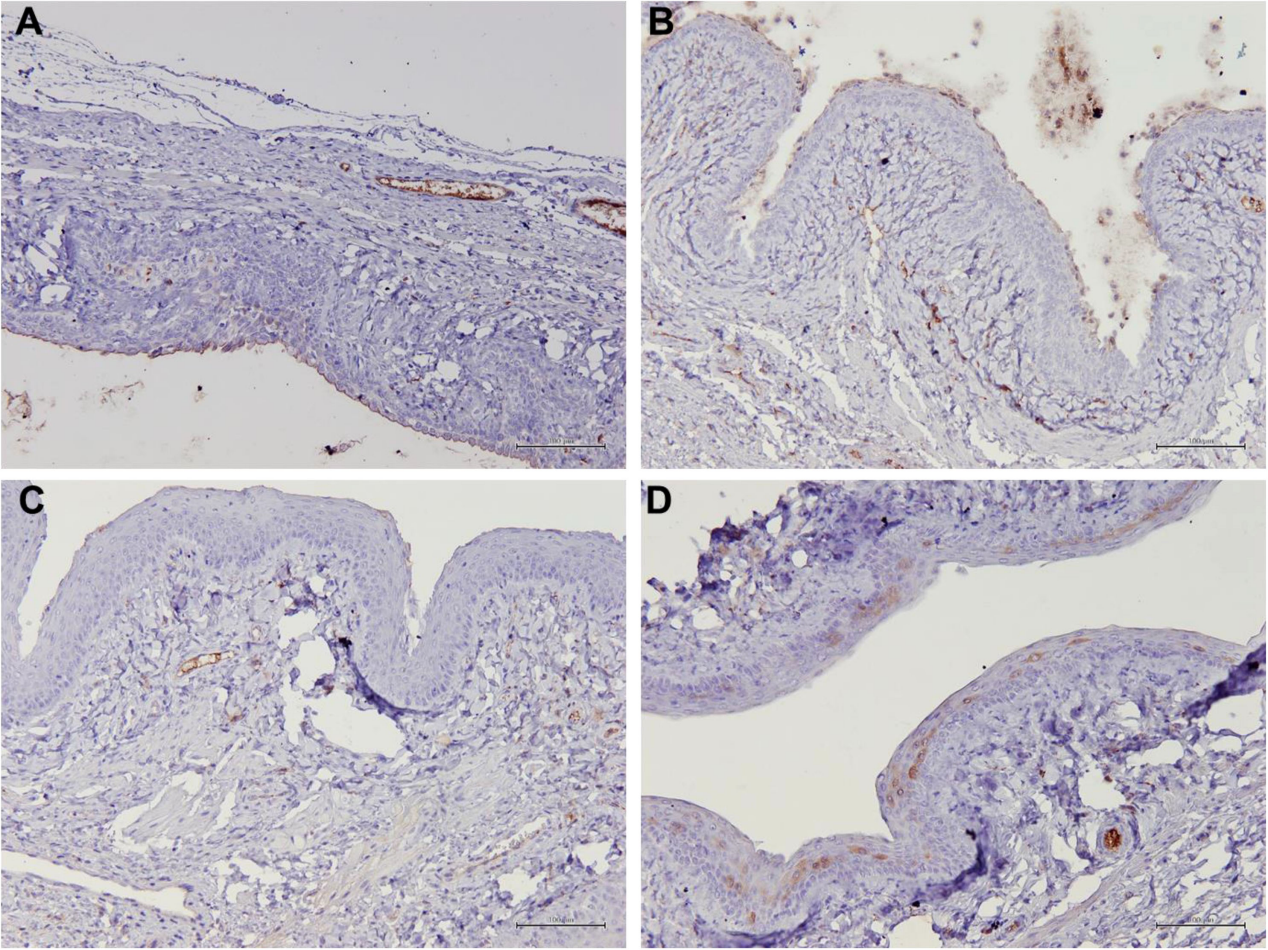
Structure of the rat vaginal epithelium and Muc1 protein localization in the rat vaginal tissue following a two-week administration of Gelam honey at 0.2 g Gelam honey/kg bw/day doses. **(A)** Gelam honey and bilateral oophorectomized group (Gelam honey +Ovx), **(B)** Estrogen replacement therapy and bilateral oophorectomized group (ERT+Ovx), **(C)** Bilateral oophorectomized group (Ovx) and **(D)** Sham group (SC). The tissues were stained with DAB/HRP stain for protein localization (in brown) and counterstained with H&E for nuclear staining. All measurement bars are equivalent to 100 µm.

**Table 1** summarizes the scoring of IHC staining for proteins Aqp1, Aqp5, CFTR and Muc1 in the vaginal tissue and its respective gene regulation level. Aqp1 was heavily localized in the vaginal tissue regardless of the treatment given to the rats. Its expression was independent of its menopause status and to an extent was also independent of the serum estrogen levels (**Figure 4**). Aqp1 localization scores ranged from 8.25 to 9 across all groups. On the other hand, expression of Aqp5 was relatively reduced in vaginal tissues (**Figure 5**). Gelam honey administration increased the localization of Aqp5 from a score of 1.25 in Ovx group to a score of 2.6 in the Gelam honey group (p <0.01). Aqp5 protein expression is directly proportional to its gene expression. CFTR protein localization was increased in the vaginal tissues of Gelam honey group (**Figure 6**). However, CFTR gene regulation was unchanged in Gelam honey +Ovx group and is only up-regulated in the ERT+Ovx group. We could not definitively conclude the relationship between CFTR gene and CFTR protein. Interestingly Muc1 protein expression was increased in the Gelam honey +Ovx group (**Figure 7**) with a score of 1.8 and exceeded the expression in ERT+Ovx group. Muc1 protein expression is directly proportional to the *Muc1* gene expression.

**Table 1:** The immunohistochemistry (IHC) scoring mean values for Aqp1, Aqp5, CFTR and Muc1 proteins in vagina epithelial tissue. The protein scoring is compared to the gene regulation data.

	Aqp1			Aqp5			CFTR			Muc1		
	IHC Score	p-value	Gene Regulation	IHC Score	p-value	Gene Regulation	IHC Score	p-value	Gene Regulation	IHC Score	p-value	Gene Regulation
Gelam honey +Ovx	9	p>0.05	–	2.6	p<0.01	˄	3	p>0.05	–	1.8	p<0.01	˄
ERT+Ovx	8.25	p<0.05	–	3.2	p<0.01	˄	2	p<0.05	˄	1	p>0.05	–
Ovx	9	p>0.05	–	1.25	p<0.01	˅	2.25	p>0.05	–	1.25	p<0.01	˄
SC	9	p>0.05	–	2.3	p>0.05	–	1.6	p>0.05	–	0.7	p>0.05	–

IHC scoring was based on a combinative semi-quantitative H-scoring method. The scoring for value A is based on percentage area of positively labelled cells (0 = 0%; 1 = below 30%; 2 = 30-60%; 4 = more than 60%). The scoring for value B is based on intensity of IHC reaction (0 = no reaction; 1 = weak reaction; 2 = mild reaction; 3 = strong reaction). The overall score presented here is the product of AxB (score range 0-9). The gene regulation is expressed as: no fold change (--); up regulated (˄); or down regulated (˅). . Gelam honey+Ovx: Gelam honey and bilateral oophorectomized group. ERT+Ovx: Estrogen replacement therapy and bilateral oophorectomized group. Ovx: Bilateral oophorectomized group. SC: Sham group

### 3.4 Assessment of atrophy in the vagina

**Figure 8:**
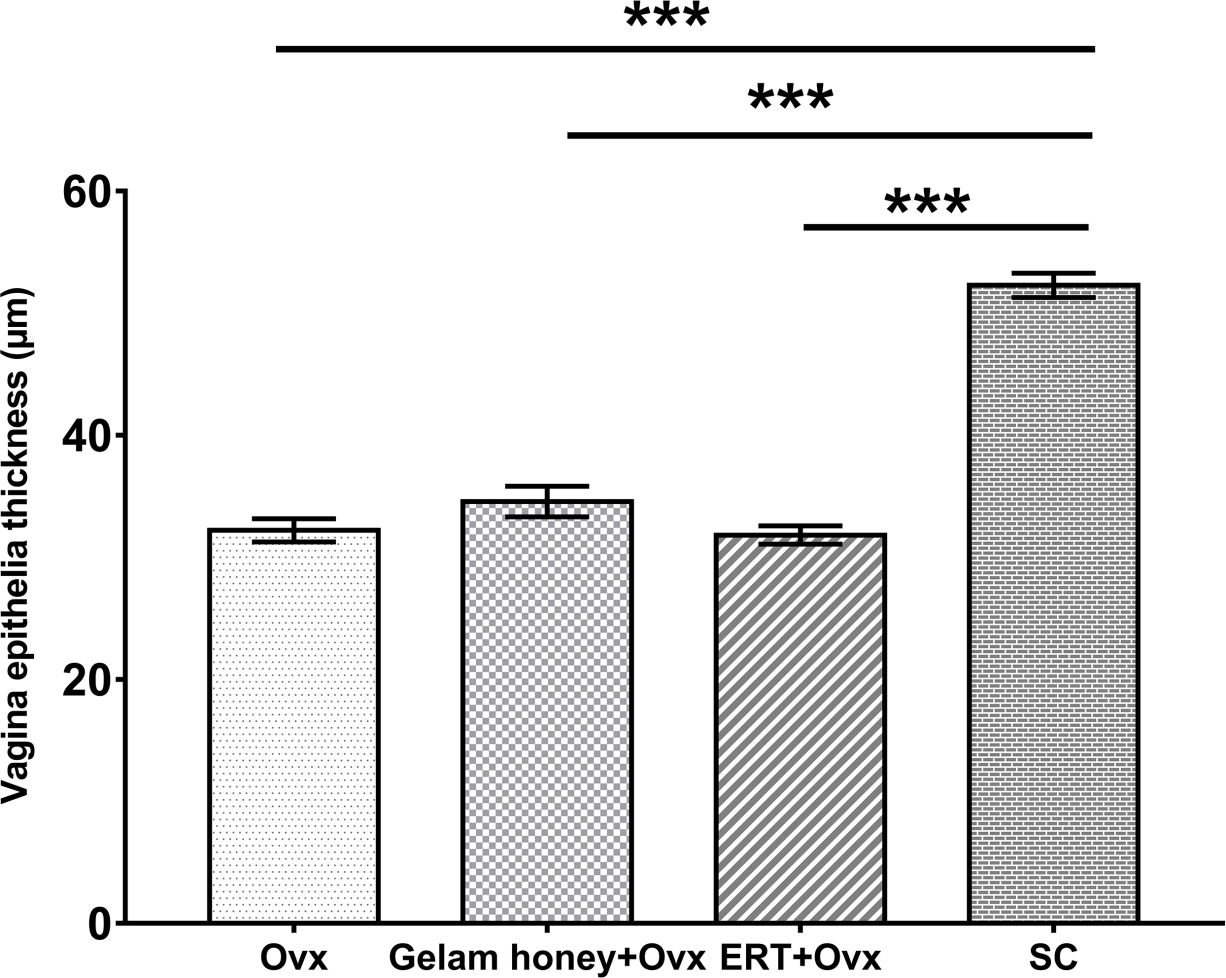
The measurements of vagina epithelia thickness (µm) for assessment of vaginal atrophy. The drying and thinning of the vagina epithelia is an indicator of atrophy. All values represent the mean ± SEM, p<0.05. Gelam honey +Ovx: Gelam honey and bilateral oophorectomized group. ERT+Ovx: Estrogen replacement therapy and bilateral oophorectomized group. Ovx: Bilateral oophorectomized group. SC: Sham group.

Analysis of vagina epithelia revealed that the epithelia thickness was adversely affected by menopause and vaginal atrophy was evident in all groups which underwent bilateral oophorectomy (**Figure 8**). The vaginal epithelia thickness for normal rats in SC group was 52.28 ± 0.99 µm. The recorded value for thickness was reduced by nearly two-folds in the Ovx group (32.21 ± 0.95 µm). However, Gelam honey was able to also attenuate the effects of estrogen deficiency caused by surgical menopause. Gelam honey revitalized the vagina epithelia thickness in Gelam honey group (34.56 ± 1.25 µm) and scored higher values than in groups receiving ERT (31.82 ± 0.75 µm). There was a statistically significant differences between groups for the mean vaginal epithelial thickness (F(3,971) = 117.31, p <0.00). The positive effects on the vagina epithelia thickness observed in the Gelam honey and ERT treatment groups was comparable. Gelam honey was able to attenuate the atrophic effects of surgically-induced menopause on the thickness of vaginal epithelia layer.This highlights the potential of Gelam honey as an alternative HRT in the treatment of menopause.

**Figure 9:**
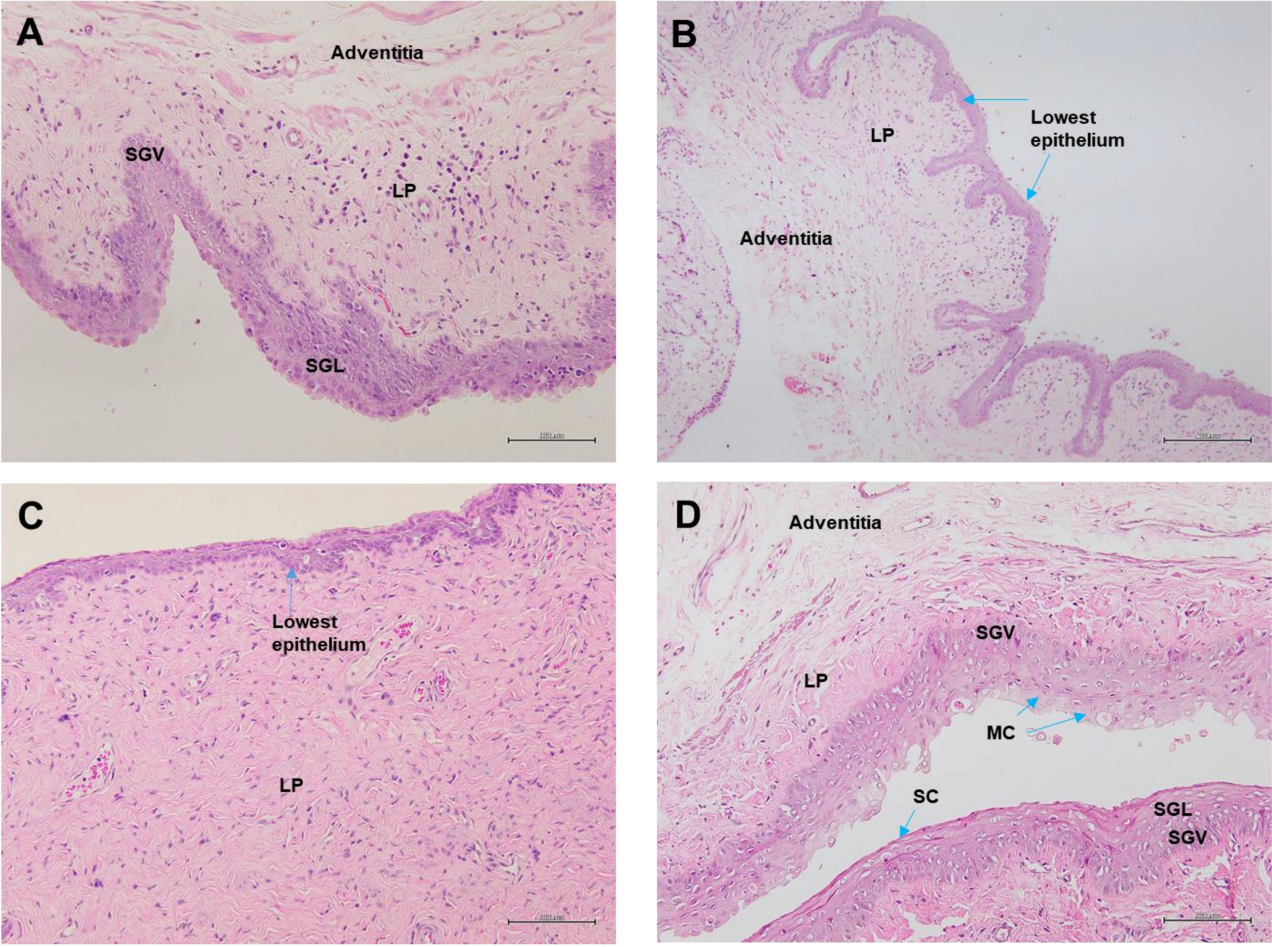
Vagina epithelia stained with H&E in **(A)** Gelam honey and bilateral oophorectomized group (Gelam honey+Ovx), **(B)** Estrogen replacement therapy and bilateral oophorectomized group (ERT+Ovx), **(C)** Bilateral oophorectomized group (Ovx) and **(D)** Sham group (SC). SGV, stratum germinativum layer; SGL, stratum granulosum layer; SM, stratum mucification layer; MC, mucus cells; LP, lamina propia; SC, startum corneum. All measurement bars are equivalent to 100 µm.

**Figure 9** shows the visualization of the vagina epithelia. The thickness of the vagina epithelia differs across all groups and is seen thickest in the Gelam honey+Ovx group. The Gelam honey +Ovx group possesses a thicker stratified epithelium with a distinct stratum germinativum (SGV) and stratum granulosum (SGL) layer. Furthermore, it was also evident that the vagina epithelia layer of the Gelam honey+Ovx group showed the presence of the stratum mucification (SM) layer with mucus cells (MC). This proves that mucous production is regained in the epithelia layer of the group receiving Gelam honey.On the contrary, there is some loss of mucoid layer of the epithelium in the ERT+Ovx group and it appears to be relatively thinner than the epithelium layer of the Gelam honey+Ovx group. The Ovx group has the thinnest epithelium layer and is devoid of the SGV layer, indicating the possibility of fewer mitotic activities within the tissue. The SC group has the thickest vagina epithelium layer composed of at least four distinct layers. The SGV, SGL, lamina propia (LP) and stratum corneum (SC) which separates the SGL from SM.

## 4 Discussion

Bilateral oophorectomy performed in this study is a procedure for the establishment of an experimental rat model with surgically-induced menopause (designated as Ovx group). Herewith, we demonstrated that surgically-induced menopause caused a significant disturbance in the levels of serum estrogen, progesterone and testosterone. It also produced a negative effect on the mRNA and protein expression levels of genes responsible for the water and mucous production in the female reproductive tract. These changes seen in the Ovx group ultimately resulted in the atrophy of the vagina, due to an abrupt cessation of secretion of the ovarian hormones. Naturally, ovarian estrogen production generally decreases as women age especially during the postmenopausal period. This condition causes the symptoms of vaginal dryness and tissue atrophy (54). The two major forms of bioactive estrogens in the normal adult female are E1 (estrone) and E2 (estradiol). E2 is predominantly synthesized in the ovaries, and in smaller amounts in the adrenal glands and adipocytes. The serum estrogen measured in this study is a measure of the E2 levels as the biological potency of E2 exceeds that of E1. Upon administration of Gelam honey in ovariectomized rats, we noted that there was a significant increase in serum estrogen level, which closely resembled the ERT treated group, as compared to the control. This finding showed that Gelam honey supplementation could benefit postmenopausal syndrome *via* its estrogenic properties.

However, the mRNA expression of Aqp1 and protein localization of Apq1 is not affected by changes in the physiological conditions of secreted estrogen levels during menopause. Incidentally, the expressions of Aqp1 is independent of estrogen levels, but is up-regulated in both groups that received either Gelam honey or ERT. On the other hand, bilateral oophorectomy affected Aqp5 by causing a down regulation, While the administration of Gelam honey and ERT increases Aqp5 mRNA expression and protein localization. However, our findings showed that ERT is more efficient than Gelam honey in modulating the changes. The protein expression is directly proportional to the mRNA expression for Aqp5 and is believed to be dependent on the secretions of estrogen. Furthermore, CFTR expression is independent of the effects of bilateral oophorectomy. ERT is able to switch on CFTR gene and upregulates CFTR expression. However, the expression of CFTR mRNA is not enough to translate into an increase in CFTR protein expression. Gelam honey administration does not exert an effect in the vagina tissue and resulted in a down-regulation of CFTR.

On the other hand, bilateral oophorectomy disrupts Muc1 regulation in which it causes the up-regulation of Muc1 gene and protein levels. However, ERT has caused a down regulation of Muc1 expression to near normal levels and is believed to have no effect on the regulation of Muc1 in the vagina. Moreover, Gelam honey has been shown to up-regulate Muc1 levels by almost a two-fold higher than normal levels. Gelam honey may achieve this due to the fructose sugar content in Gelam honey that contributes to the sialic acid synthesis as fructose is a preferred substrate (55), thus leading to an accumulation of mucins in the tissue. Muc1 is also estrogen dependent, and the estrogenic properties of Gelam honey is likely to have contributed to the upregulation. Traditionally, an assumption that was commonly made was mRNA expressions can be indicative of predictions of the corresponding protein expressions (56, 57). This is true for the mRNA and protein expressions of Aqp5 and Muc1. However, the discordance between the mRNA and protein expressions of Aqp1 and CFTR proves otherwise. This could be attributed to the fact that different proteins expressed by the same cell may possess distinctive glycosylation patterns (58).

Vagina epithelia thickness greatly depends on the normal secretion of estrogen (59). The surgically-induced menopause adversely affected the thickness of the vagina epithelium. The two-week administration of Gelam honey or ERT was unable to fully overcome the effects of surgically-induced menopause on the thickness of the vagina epithelia to normal levels. However, Gelam honey has a slight advantage and has been shown to be as effective as ERT in regaining the thickness of the vagina epithelia. Similar increase in thickness of the vagina epithelia was also reported by Zaid et al. (60) when using Tualang honey. It is believed the cell proliferation effect is attributed by flavonoid components of honey that are known for their weak estrogenic properties. Major flavonoids in honey with notable estrogenic property are kaempferol and quercetin and are examples of phytoestrogens (61).

In conclusion, Gelam honey down-regulated CFTR expression and concurrently, up-regulated the expression of Aqp1, Aqp5 and Muc1. Based on the gene and protein expression results, Gelam honey can maintain water transport within the vagina tissue and maintain an intact mucous layer on the vagina epithelial layer. The effects of Gelam honey on the serum estrogen and vagina epithelium’s thickness are comparable to or is (in the case of vagina epithelium thickness) better than the effects of ERT. Gelam honey administration attenuates vaginal atrophy in cases of surgically-induced menopause. Gelam honey is a conceivable candidate for an alternative HRT attributable to its estrogenic properties and could be a viable option in cases of HRT upon surgically-induced menopause.

## Data availability statement

The original contributions presented in the study are included in the article/**Supplementary Material**. Further inquiries can be directed to the corresponding author/s.

## Ethics statement

The animal study was reviewed and approved by Institutional Animal Ethics Committee of the Universiti Kebangsaan Malaysia (UKMAEC) for animal research (approval no.: FISIO/PP/2019/SITI FATIMAH/30-OCT./1059-OCT.-2019-APR.-2022-NAR-CAT2).

## Author contributions

NHI wrote the manuscript, performed the investigations, collected data and performed formal analyses for the immunohistochemistry assays for the protein localization study and qPCR experimental design and assays for gene expression analysis. SJA assisted in conducting the gene expression study and contributed to writing the introduction. AFZ and NHI performed the bilateral oophorectomy surgery. AY and NHI conducted the immunohistochemistry scoring and interpretation of tissue sections. MHM provided the project administration and the research environment. SFI and KO conceptualized, supervised and validated the study. NHI, SFI and KO reviewed and edited the manuscript. MHM, NHI, SFI and KO wrote and edited the grant supporting the work. All authors contributed to the article and approved the submitted version.
