# Supplementary material for: Modulation of vulvovaginal atrophy (VVA) by Gelam honey in bilateral oophorectomized rats
Source: Front Endocrinol (Lausanne). 2023 Feb 27;14:1031066. doi: 10.3389/fendo.2023.1031066 (PMC10010262; doi:10.3389/fendo.2023.1031066)
Supplement: Supplementary file 1 [file Table_1.docx]

| **Gene** | **GenBank#** | **Forward primer** | **Reverse primer** |
| --- | --- | --- | --- |
| Aqp1 | NM_012778 | TATATCATCGCCCAGTGTGTG | GTGCCAATGATCTCAATGCC |
| Aqp5 | NM_012779 | CTCCCCAGCCTTATCCATTG | CCTACCCAGAAGACCCAGT |
| CFTR | NM_031506 | CCTTCGATATTTCACGCTCCA | GCCATTGTTTCCACCATTAACG |
| Muc1 | NM_012602 | CCATCCTATGAGTGAATATCCTACC | GTGTAAGAGAGACCGCTACTG |
| β-Actin | NM_031144 | TCACTATCGGCAATGAGCG | GGCATAGAGGTCTTTACGGATG |
| Gapdh | NM_017008 | AACCCATCACCATCTTCCAG | CCAGTAGACTCCACGACATAC |

**TABLE S1|** The primer sequence for Quantitative Real-Time PCR (RT-qPCR)
